# Analysis of the Anti-Vaccine Movement in Social Networks: A Systematic Review

**DOI:** 10.3390/ijerph17155394

**Published:** 2020-07-27

**Authors:** Elvira Ortiz-Sánchez, Almudena Velando-Soriano, Laura Pradas-Hernández, Keyla Vargas-Román, Jose L. Gómez-Urquiza, Guillermo A. Cañadas-De la Fuente, Luis Albendín-García

**Affiliations:** 1 Faculty of Health Sciences, University of Granada, 18016 Granada, Spain; eosanchez@correo.ugr.es (E.O.-S.); jlgurquiza@ugr.es (J.L.G.-U.); gacf@ugr.es (G.A.C.-D.l.F.); 2 Andalusian Health Service, Virgen de las Nieves University Hospital, 18014 Granada, Spain; 3 Andalusian Health Service, San Cecilio Clinical University Hospital, 18016 Granada, Spain; lauraphl9@gmail.com; 4 Faculty of Psychology, University of Granada, 18071 Granada, Spain; keyvarom@ugr.es; 5 La Chana Health Center, Granada Metropolitan District, Andalusian Health Service, 18015 Granada, Spain; lalbendin@yahoo.es

**Keywords:** vaccines, social networks, false information, internet, parents, children

## Abstract

The aim of this study was to analyze social networks’ information about the anti-vaccine movement. A systematic review was performed in PubMed, Scopus, CINAHL and CUIDEN databases. The search equations were: “vaccine AND social network” and “vaccine AND (Facebook[title] OR Twitter[title] OR Instagram[title] OR YouTube[title])”. The final sample was *n* = 12, including only articles published in the last 10 years, in English or Spanish. Social networks are used by the anti-vaccine groups to disseminate their information. To do this, these groups use different methods, including bots and trolls that generate anti-vaccination messages and spread quickly. In addition, the arguments that they use focus on possible harmful effects and the distrust of pharmaceuticals, promoting the use of social networks as a resource for finding health-related information. The anti-vaccine groups are able to use social networks and their resources to increase their number and do so through controversial arguments, such as the economic benefit of pharmaceuticals or personal stories of children to move the population without using reliable or evidence-based content.

## 1. Introduction

The rejection of immunization by the population was born with the creation of the first vaccine in 1796 when Jenner presented protection against smallpox to the Royal Society of London. From this point, a rise in England’s first compulsory vaccination through a strong campaign began to appear unhappy [1], which led to the constitution of the League against Mandatory Vaccination of London in 1867, and it began to spread this movement to the rest of Europe [2].

This movement won strength in 1998 when Wakefield published an article in “The Lancet”, in which he related the possibility of suffering autism with the administration of the vaccine against rubella, mumps and measles. This sonorous article caused a 9% drop in the vaccination rate in the United Kingdom in just one year. In the end, it was proved that Wakefield and the co-authors of the article had conflicts of interest and the journal was forced to publish a retraction but, despite that, this belief is still maintained today [3].

Currently, a study by the American Academy of Pediatrics reveals that 74% of pediatricians find parents who oppose or have delayed the administration of vaccines to their children. Another survey of parents with children aged 6 months to 6 years shows that 13% opted for an alternative immunization program, 53% rejected one vaccine and 17% rejected all [4].

The anti-vaccination groups base their arguments on their lack of confidence in the information provided by health professionals and official sources about vaccines. Generally, they have doubts about the administration of multiple vaccines at such early ages and the lack of individualization of these drugs. Their fear lies in the possible adverse effects and the constant change in the vaccination schedule, as well as in the differences between autonomous communities. This is linked to the belief that because the disease has very low incidence it is not necessary to vaccinate their children (which is, in fact, due to the vaccine) or because they believe in natural remedies or alternative medicine, so people in the anti-vaccine group end up looking for information that confirms their beliefs [5,6]. In addition, it shows that people who refuse vaccines are more likely to obtain information from social networks, not from health professionals or verified healthcare websites [6]. Another study informs that 52% of people who use the Internet consider this medium reliable in terms of health issues [7].

Despite the fact that healthcare professionals remain the main source of health information, including vaccines, the Internet has grown as a resource for finding information due to its high accessibility [8]. In fact, vaccine-related searches on the Internet have increased, most of them coinciding with the start of influenza vaccination campaigns [9]. The increase of web information search causes an opportunity for the appearance of websites with unreliable content generating false beliefs [10].

Considering the importance that the anti-vaccine movement has gradually acquired, the aim of this study was to analyze social networks’ information about the anti-vaccine movement. Thus, the PICO question that guided the review was: Which information (O) does the anti-vaccine movement (P) use in social networks (I) to influence the global population (C)?

## 2. Materials and Methods

A systematic literature review was conducted following the PRISMA guideline. No protocol was registered.

### 2.1. Eligibility Criteria

We included primary studies related to the use of social networks and the anti-vaccine movement that collect samples on the main social networks (Twitter, Facebook, Instagram and YouTube), published in English and Spanish and conducted over the last 10 years.

All articles were exclusively related to the measles outbreak and the HPV vaccine; articles using samples not obtained on Twitter, Facebook, Instagram or YouTube and articles without statistical information and duplicate articles were excluded.

### 2.2. Information Sources and Search

The databases used were CINHAL, CUIDEN, PubMed (Medline) and Scopus. The first search equation based on MeSH terms was “Vaccine and social networking”. The second search equation was “vaccine AND (Facebook[title] OR Twitter[title] OR Instagram[title] OR Youtube[title])”. The search equations were adapted to each database. The search was conducted in December 2019. In addition, a reverse search was performed in the selected studies. 

### 2.3. Study Selection and Risk of Bias

The selection of the studies was done independently by two researchers and had 4 phases. Firstly, title and abstract were read. Secondly, the full text was read. Then, a reverse search was done with the included studies to locate as many documents as possible; and finally, a critical reading of the studies was carried out to evaluate possible biases in the methodology. The SIGN classification (2011) was used to provide the level of evidence.

### 2.4. Data Collection, Variables and Data Analysis

A data collection notebook was used to extract the data from each study. The following data were collected from each study:

1. Variables about the characteristics of the sample: year of publication, country of study, study design, number of tweets, Facebook or Instagram comments or YouTube videos.
2. Variables about the study: aim and main results of each article.

The analysis of the data was descriptive.

## 3. Results

### 3.1. Study Selection

**Figure 1:**
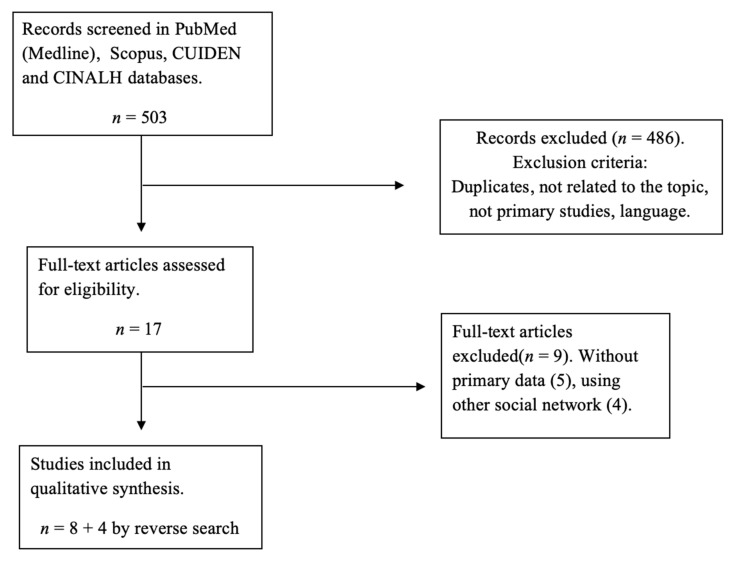
Flow diagram of the study selection.

A total of 503 articles were obtained from the database search as of December 2019. After reading the title and abstract, 486 studies were excluded for not meeting the inclusion criteria or being duplicates. After reading the full text of the remaining articles, eight were excluded for not meeting the inclusion criteria. So, eight articles from the search were included in the review. Finally, four studies were included after the reverse search, leaving a sample of *n* = 12 articles. The flow chart with the study selection process is shown in Figure 1.

### 3.2. Study Characteristics

All selected articles were descriptive cross-sectional studies. The evidence level according to SIGN [11] evidence scale was 3D. The 41.66% of the articles were published in 2019, 50% show data obtained on Twitter and 50% have been made in the United States. The 12 articles included showed data on three social networks: YouTube, Twitter and Facebook. Half of the studies (50%) were based on Twitter. After the analysis, two categories of results were established.

### 3.3. Twitter and Vaccine Information

The study by Gunaratne et al [12] shows an increase in Twitter content in favor of vaccines; despite this, it highlights an increase in users defending the anti-vaccine movement. This information is reinforced in the study led by Blankenship et al [13], which highlights that everything related to the anti-vaccine group has a greater number of interactions, retweets or likes. 

Another study indicates that approximately 12% of websites with vaccine content shared on Twitter have low credibility [14] and use a different language between those webs that are in favor and those that are against vaccines. For example, those that favor vaccines refer to all stages of life, while the anti-vaccine refer only to childhood. In addition, the type of words most often used by websites against vaccine are names of diseases that may have been related to the administration of vaccines, such as autism, relating it to vaccine components [15]. This is supported by the study of Love et al. [16], which determines that tweets against vaccines focus on the supposed harm that they cause. Twitter also has users called “trolls” or “bots” that generate more content about vaccines than a normal user, being mostly against them [17]. All studies agree that the mechanisms to spread the anti-vaccine message in Twitter are the use of personal stories, talking about the risks of vaccines and their components, the business of the pharmaceutical industry and conspiracy theories, sometimes supported with links to websites based on no evidence [12,13,14,15,16,17]. Nevertheless, pro-vaccine tweets and users have more presence on Twitter than anti-vaccine tweets and users [12,13,14,15,16,17].

### 3.4. Facebook and YouTube and Vaccine Infringement

Facebook users who showed negative feelings about vaccines are introduced as a “pro-science” group that tries to give information about vaccines that is supposedly being hidden [18]. Tustin et al. [19] revealed that the comments of this social network mostly speak about distrust towards pharmacists or healthcare providers and include negative experiences with vaccines. These studies are based on the new advertising tool from Facebook, in which anti-vaccination ads have been included talking about alleged institutional fraud and promoting vaccination [20]. In terms of interaction, comments in favor of immunization receive more “likes” than those against [21], although the latter group consumes more content [22]. As on Twitter, antivaccine users based their posts and comments on personal stories, vaccines risks, vaccine components, distrust in pharmaceutical industry and conspiratory theories [18,19,20,21,22]. Even though pro-vaccine users and posts have more presence, anti-vaccine users seems to grow more cohesively on Facebook than pro-vaccine groups [22].

On YouTube the most watched videos about vaccines are about personal stories that had more views than videos by health agencies. In addition, the search terms are similar for videos presented for and against vaccines [23].

Table 1 summarizes the information from the included studies.

## 4. Discussion

After the literature review, it was observed that Twitter seemed to be the most used social network by the anti-vaccine movement. The anti-vaccine users are fewer than pro-vaccine, but they are more active. The anti-vaccine groups usually use the same reasons in their tweets or posts (vaccine risks, autism, vaccine component and conspiracy theories) and base their speech on personal stories. This is linked to the distrust of pharmaceuticals by the anti-vaccine group [17], which is based on the belief that they have great economic gains from vaccines, having no evidence for the information that they disseminate [5,24]. They also argue that because there is no incidence of some diseases, there is no need for vaccination; but the low incidence of some diseases is due to vaccines [6,8].

It is also interesting to see how people who are against immunization use words in their language related to vaccine components such as "mercury", inciting users’ distrust [22]. This is due to the rejection of chemical products for fear of suffering health problems [25] and the preference for alternatives with natural products by the anti-vaccine group [5]. This movement is mainly growing in western countries partly due to unlimited access to health-related information on the Internet [26]. 

Another example of the spread of false information on social networks can be seen with the recent SARS-Covid-19 pandemic that we are suffering. The novelty of the disease causes false news of both its origin and its treatment to spread rapidly. One of the most popular formulas has been to mix sodium chlorite with citric acid as a treatment against the virus, a remedy that has no evidence [27,28]. This type of news can confuse the population, as well as be dangerous to their health, as is the case with vaccines. Moreover, as it happens with other vaccines, some false information is growing on the Internet and social networks with the COVID-19 pandemic, with some groups saying that the virus does not exist or that future vaccine will have a microchip to control us [29,30]. Thus, even with the danger of the pandemic, the anti-vaccine movement is still there. The trend and future of this movement depend on the efforts of healthcare professionals, health organizations and social networks to prevent fake information dissemination which is the main technique that they use to hook people [31,32]_._ Without any intervention, surely this movement will grow.

The influence of the anti-vaccine movement on social networks can be prevented with strategies that are already working, like creating social networks accounts for official health organizations or modifying the search logarithm of social networks, showing the official information from verified sources first when a user looks for information about vaccines or the COVID-19 pandemic (as it is being done on the main social networks) [33,34]. Furthermore, a strong emphasis on parents’ education about how to find and trust checked information in health institutions should be taken into account [26].

This study shows the need for greater training for the population to learn how to detect fake information and, on the part of health agencies, to be attentive to possible misinformation that may arise and refute it with true and accessible data in a simple language accessible to the population. They should also promote strategies to try to reach more people on the net to combat misinformation and fake news.

### Limitations

This study has some limitations. First, the number of articles is low, mainly because the use of social networks to find health-related information is a recent phenomenon. Differences between countries should also be taken into account in the interpretation of the results, as some have less access to the Internet or its influence on the population is lower. Also, some terms related to the topic were not included in the search equation. Future research could analyze this movement against concrete vaccines, like the HPV, or to analyze how training courses on fake information detection can influence the beliefs of the population.

## 5. Conclusions

Anti-vaccine groups are using social networks to spread health information, creating their own content without any evidence to confuse users who access their pages. To do this, most of the time they use alleged stories about children who have suffered side effects that end up moving the readers—a fact that impacts more than the scientific data provided by health agencies.

Another method used by the anti-vaccine groups to attract different users is the debate generated by bots and trolls about vaccination. They create contentious debates arguing that pharmaceutical companies make a profit. The methods used by groups against vaccination on social networks are diverse and in many cases are useful for their task.

## Figures and Tables

**Table 1:** Summary of included studies (*N* = 12).

Author, Year, Country of Study.	Study Design	Sample Size	Objective	Main Results	Level of Evidence/Degree of Recommendation
**Gunaratne et al, 2019, Canada. [12]**	Descriptive cross-sectional study.	1,637,712 tweets	To reflect the temporal trends of discussions for and against vaccines on Twitter and determine the extent of communication between the two groups.	The study reveals an increase in speeches in favor of vaccines from 2014. Despite this, the opposite group continues to grow in size, and communication between both is minimal. 576,695 tweets (35%) were anti-vaccine. The hashtags #cd-cwhistleblower, #vaxxed, #hearthiswell, #novax and #cd-cfraud were the most used by the anti-vaccines groups. From 291,747 users, 12% posted only anti-vaccine hashtags, increasing from 8.1 in 2015 to 16% in 2018.	3/D
**Blankenship et al, 2018, United States. [13]**	Descriptive cross-sectional study	1545 tweets	To investigate the level of participation that tweets with different opinions about vaccines attract.	They analyzed two hashtags: #vaccine and #vaccineworks selecting a random sample. From 1344 analyzed tweets with the hashtag #vaccine, 24.2% were about the anti-vaccine sentiment and 59.1% of them had links to websites. Anti-vaccine tweets were focused on risk and dangers and distrust of pharmaceutical industry, science or government. The 201 tweets with the hashtag #vaccineswork did not have information about anti-vaccine movement. Tweets against vaccines were more likely to interact (retweets) than those expressing feelings for them. These two, in addition, had greater participation than the tweets that were neutral.	3/D
**Shah et al, 2019, Australia. [14]**	Descriptive cross-sectional study.	6,591,566 tweets	To characterize the potential scope of shared vaccine websites on Twitter in relation to credibility.	Among shared websites, 11.86% maintained low credibility and generated 112,225 retweets (14.68%). Of these, it is estimated that 100 Most Viewed were visited by between 2 and 10 million Twitter users. The low-credibility web pages linked in twitter were related to individual stories and autonomy.	3/D
**Kang et al, 2017, United States [15]**	Descriptive cross-sectional study.	50 websites shared on Twitter.	To know the opinion about vaccines of Twitter users through the semantics of the web pages they share.	Of the web pages shared, 23 pages showed positive feelings towards vaccines, 21 were negative and 6 were neutral. The pages that spoke positively treated it from the point of view of childhood, adolescence and adults, but the negative ones only talked about childhood. The most commonly used concepts in the negative pages were: children, mercury, autism, industrialization of vaccines, ingredients of vaccines.	3/D
**Love et al, 2013, United States. [16]**	Descriptive cross-sectional study.	2580 tweets.	To analyze the content provided by Twitter on vaccination, taking into account the medical reliability to know the type of information about which users are interested and thus lead educational campaigns.	Of the tweets analyzed, 33% were positive for immunization, 54% maintained a neutral position and 13% were against. Those in favour made contributions promoting their administration, the neutrals reported experiences on the subject and the negatives consisted of claims about vaccine damage.	3/D
**Broniatowski et al, 2018, United States. [17]**	Descriptive cross-sectional study.	899 tweets from bots and trolls and 9895 tweets from actual Twitter users.	To understand how Russian bots and trolls promote health content on Twitter.	Accounts identified as trolls or bots were more likely to tweet about vaccine content than normal users and were less likely to create preventable disease content. Bot accounts tended to post more anti-vaccine content than normal users but their message was less polarized. From the analysis of 253 tweets with the hashtag #vaccinateUS, 38% were anti-vaccine. These were usually related to conspiracy theories and risks.	3/D
**Hoffman et al, 2019, United States. [18]**	Descriptive cross-sectional study.	197 Facebook users	To systematically evaluate people who express negative feelings related to vaccines on Facebook.	Most of them were female (89%) and parents (78%). These publications were taken from anti-vaccination groups that referred to themselves as "pro-information" or “pro-science”. Their anti-vaccination posts were about their risks and damages, indicating that vaccines caused diseases and death, personal stories and conspiracy theories.	3/D
**Tustin et al, 2018, Canada. [19]**	Descriptive cross-sectional study.	117 comments from Facebook	To analyze the content of Facebook users’ comments to find out the main feelings towards vaccinations.	Of the 85 commentators 77% were female. Of all comments about vaccinations, 43.6% were positive, 35% negative and the rest were ambiguous. Negative comments included misperceptions of risk, inaccurate knowledge, distrust of pharmacists or health care providers, negative experiences with vaccines or beliefs. Almost 40% of positive reviews spoke of the risks of non-vaccination and judged the level of knowledge of anti-vaccination.	3/D
**Jamison et al, 2019, USA. [20]**	Descriptive cross-sectional study.	309 vaccine-related ads on Facebook	To analyze the new Facebook tool to publish ads and the reliability of these related vaccines.	Of all ads, 53% were pro-vaccines and 47% anti-vaccine. However, only 27 people were anti-vaccine ad buyers, while 83 were pro-vaccine. The pro-vaccine announcements were divided into five themes: vaccine promotion, philanthropic work, advocacy of vaccination policies, news and anti-vaccine views. Anti-vaccines were more unified, described the damage done, promoted the choice of vaccine and revealed an alleged institutional fraud.	3/D
**Faasse et al, 2016, Australia. [21]**	Descriptive cross-sectional study.	1489 comments from Facebook.	To investigate the language used by people for and against immunization in the same conversation in order to provide an optimal education.	Comments in favor of vaccines received more "likes" than those against and neutrals, the latter being the least receiving of the three. The positive comments were more truthful than the negative ones and these in turn contained less family-related words than the other two groups. Anti-vaccination comments were based on risks and causation words and fewer positive emotion words. They also had lower authenticity and references.	3/D
**Schmidt et al, 2018, Italy. [22]**	Descriptive cross-sectional study.	243 Facebook pages.	To assess whether the conduct of users about immunization is polarized and how it evolves over time.	Most users were active in one group, for or against vaccines, but not in both. The anti-vaccine group consumed information from a more diverse set of pages than pro-vaccine. Anti-vaccine were more committed to their post consumption. The ant-vaccine community grew in a more cohesive manner on the social network, with less fragmentation.	3/D
**Yiannakoulias et al, 2019, Canada. [23]**	Descriptive cross-sectional study.	206 YouTube videos	To report strategies that increase useful information on YouTube regarding pro and anti-vaccine content.	The most frequent searches were about personal stories rather than about the benefits or how vaccines worked, so videos from public health agencies had fewer views. In addition, the search terms are very similar for both pro-vaccine and anti-vaccine content. Anti-vaccine videos contained more target words and had higher likeability. The words mercury, syringe, cheical and toxic were more used in anti-vaccine videos.	3/D
